# Supplementary material for: Field Evaluation of Wheat Varieties Using Canopy Temperature Depression in Three Different Climatic Growing Seasons
Source: Plants (Basel). 2022 Dec 12;11(24):3471. doi: 10.3390/plants11243471 (PMC9785455; doi:10.3390/plants11243471)
Supplement: Supplementary file 1 [file plants-11-03471-s001.zip › supplementary/Supplement-Tables 20221105.pdf]

Supplementary Table 3. Descriptive statistics of canopy structural traits in three growing seasons.

**Traits**	**2016-17(Normal)**		**2017-18(Freezing)**		**2018-19(Drought)**	
	**Mean±SD**	**CV**	**Mean±SD**	**CV**	**Mean±SD**	**CV**
WFL (cm)	1.92±0.2	10.42%	1.72±0.18	10.47%	1.8±0.19	10.56%
LFL (cm)	19.83±2.5	12.61%	14.29±1.75	12.25%	16.48±2.48	15.05%
FLA (cm^2)	42.51±17.76	41.78%	29.02±11.85	40.83%	40.66±12.5	30.74%
PH (cm)	85.63±8.62	10.07%	66.54±7.28	10.94%	74.13±9.33	12.59%
PL (cm)	27.6±3.56	12.90%	23.87±3.71	15.54%	23.25±3.94	16.95%
DSL (cm)	10.38±2.95	28.42%	9.12±3.21	35.20%	7.18±3.1	43.18%

Note: the data was shown as the Mean ± standard deviation, and the coefficients of variations (CV) was also shown; WFL: Width of flag leaf; LFL: Length of flag leaf; FLA: Flag leaf area; PH: Plant height; PL: Peduncle length; DSL: Distance of spike to leaves.

Supplementary Table 4. Variations of yield traits in the three wheat growing seasons.

Growing season	Index	GPS	SN (m^{-2})	TKW (g)	GY (kg/hm^{-2})	BM (kg/hm^{-2})	HI
2016-17 （Normal）	Mean±SD	47.2±7	710±109	39±4.9	8744±1652	21916±3463	0.41±0.05
	CV	14.83%	15.36%	12.56%	18.89%	15.80%	12.20%
2017-18 (Freezing)	Mean±SD	38.8±6.8	356±79	47.9±4.4	6941±934	15158±2205	0.42±0.07
	CV	17.53%	22.10%	9.19%	13.46%	14.55%	16.67%
2018-19 (Drought)	Mean±SD	45.5±6.6	438±90	48.1±4.6	7388±1368	15023±2964	0.47±0.05
	CV	14.51%	20.50%	9.56%	18.52%	19.73%	10.64%

Note: the data was shown as the Mean ± standard deviation, and the coefficients of variations (CV) was also shown; GPS: grains per spike; SN: spike number per m^2; TKW: 1000-kernel weight; GY: Grain yield; BM: biomass aboveground; HI: Harvest index

Supplementary Table 5. Comparisons of CTD among the clusters of wheat varieties based on CTDs at three growth stages for each growing season.

Growing season	Cluster	No. of varities	HD (℃)	EF(℃)	MF(℃)
			Mean ± SD	Mean ± SD	Mean ± SD
2016-17	1	79	-1.5±0.64 a	-0.9±0.63 a	-1.7±1 a
（Normal）	2	64	-3±0.69 b	-1.7±0.61 b	-4.4±0.86 b
	3	43	-4.7±0.82 c	-3.6±1 c	-4.9±0.87 c
2017-18	1	27	-2.3±0.42 a	-2.1±0.46 b	-0.76±0.48 a
(Freezing)	2	70	-3.4±0.74 b	-1.9±0.48 a	-1.8±0.57 b
	3	82	-3.8±0.56 c	-2.8±0.51 c	-2.6±0.62 c
2018-19	1	79	-0.5±0.62 a	-5.4±0.51 a	-1.1±0.65 a
(Drought)	2	78	-1.9±0.62 b	-5.6±0.71 b	-2.4±0.77 b
	3	29	-3.2±0.71 c	-6.8±0.5 c	-3±0.5 c

Note: the data was shown as the Mean ± standard deviation. The clustering was conducted with the CTD at the three growth stages for each growing season using the Ward method. Multiple comparison among the averages of the three clusters for each stages was conducted using the Duncan's test, different lowercase letters indicated the differences significant at the $P < 0.05$ level. HD: Heading; EF: Early filling; MF: Middle filling.

Supplementary Table 6. Multiple comparisons of photosynthetic traits among the clusters of wheat varieties based on CTDs at the three stages for each growing season.

Growing season	Stage	Cluster	CTD (°C)	A (μmol m^{-2} s^{-1})	Gs (μmol m^{-2} s^{-1})	Ci (μmol mol^{-1})	E (mol m^{-2} s^{-1})	A/E	Ci/Ca
2016-17 (Normal)	HD	1	-1.5±0.64 a	21.2±2.2	0.34±0.06	254.1±21.3 a	3.8±0.51 b	5.7±1 a	0.68±0.05 a
		2	-3.0±0.69 b	21.8±2.3	0.34±0.09	240.4±30.2 b	4.1±0.75 a	5.4±0.88 ab	0.65±0.08 b
		3	-4.7±0.82 c	21.7±2.4	0.33±0.07	245.2±18.4 b	4.2±0.59 a	5.2±0.51 b	0.65±0.05 b
	EF	1	-0.9±0.63 a	20.6±2.3 a	0.27±0.06	230.6±25.4	5.2±0.72 a	4.0±0.57 b	0.61±0.07
		2	-1.7±0.61 b	18.9±2.3 b	0.26±0.05	237.1±24.8	4.4±0.75 b	4.4±1.1 a	0.63±0.07
		3	-3.6±1.0 c	18.7±2.0 b	0.26±0.06	233.5±20.5	4.4±0.76 b	4.3±0.75 a	0.63±0.06
	MF	1	-1.7±1.0 a	8±2.8 c	0.33±0.08 a	342.1±16.7 a	4.4±0.82 a	2.6±1.1 a	0.87±0.04 a
		2	-4.4±0.86 b	10.8±3.4 b	0.34±0.08 a	325.2±23 b	4.2±0.8 a	2.5±0.8 b	0.83±0.05 b
		3	-4.9±0.87 c	12.5±5.2 a	0.27±0.07 b	297.1±45.1 c	3.7±1 b	2.6±1.1 a	0.76±0.1 c
2017-18 (Freezing)	HD	1	-2.3±0.42 a	15.7±2 c	0.26±0.06 b	261.9±20.3 b	4.4±1 b	3.7±0.7 a	0.68±0.05 ab
		2	-3.4±0.74 b	19.2±3.5 a	0.33±0.08 a	264.4±17.3 b	6±1.3 a	3.3±0.6 b	0.69±0.04 b
		3	-3.8±0.56 c	17.4±3.5 b	0.33±0.07 a	273.1±19.6 a	6.2±1.4 a	2.9±0.7 c	0.71±0.05 a
	EF	1	-2.1±0.46 b	18.6±1.8	0.29±0.06 b	281.1±25 b	4.2±1 b	4.7±1.1 a	0.7±0.05 b
		2	-1.9±0.48 a	17.6±2.3	0.32±0.05 a	297.5±25.6 a	5.3±1 a	3.5±1 b	0.74±0.05 a
		3	-2.8±0.51 c	18.1±2.2	0.31±0.05 ab	290.6±19.2 ab	5.7±0.9 a	3.3±0.6 b	0.73±0.04 a
	MF	1	-0.76±0.48 a	16.2±2.2 ab	0.19±0.05 a	235±22.7	2.3±0.6 b	7.5±2.2 a	0.6±0.05
		2	-1.8±0.57 b	17.2±2.3 b	0.22±0.05 b	236.1±22.1	3.1±0.9 a	6±1.5 b	0.61±0.06
		3	-2.6±0.62 c	17.7±2.5 a	0.24±0.09 ab	240±25.6	2.9±0.7 a	6.3±1.1 b	0.62±0.08
2018-19 (drought)	HD	1	-0.5±0.62 a	30.1±3.5 a	0.53±0.09 a	288.8±14.7 b	9.2±1.5 a	3.3±0.38 ab	0.72±0.04 a
		2	-1.9±0.62 b	26.1±3.4 b	0.36±0.15 b	241.7±55.6 a	7.7±1.4 b	3.5±0.43 a	0.6±0.14 b
		3	-3.2±0.71 b	24.4±2.7 c	0.29±0.12 c	226±50.1 a	7.8±1.1 b	3.2±0.45 b	0.57±0.13 b
	EF	1	-5.4±0.51 a	28.9±3.1 a	0.51±0.1 a	288.6±16.1 a	8.2±1.4 a	3.6±0.47 b	0.72±0.04 a
		2	-5.6±0.71 b	26.3±4.9 b	0.43±0.1 b	282.5±18.9 ab	6.8±1.5 b	4±0.92 a	0.71±0.05 ab
		3	-6.8±0.5 c	28.1±3.9 a	0.45±0.08 b	278.4±11.1 b	6.8±1.1 b	4.1±0.39 a	0.7±0.03 b
	MF	1	-1.1±0.65 a	22.8±2.4	0.37±0.08 a	276.1±19 a	5.1±1 a	4.6±0.81 b	0.7±0.05 a
		2	-2.4±0.77 b	22.1±2.2	0.32±0.06 b	268.1±18.3 ab	4.5±0.77 b	5±0.77 a	0.68±0.05 b
		3	-3±0.5 c	21.9±1.5	0.3±0.06 b	262.2±19.5 a	4.2±0.74 b	5.3±0.82 a	0.67±0.05 b

Note: the data was shown as the Mean ± standard deviation. the clustering was conducted with the CTD at the three growth stages using the Ward method. Then multiple comparison was conducted among the three clusters for each stage using the Duncan method, different lowercase letters indicated the differences significant at the $P < 0.05$ level. A: Net photosynthetic rate; E: Transpiration rate; Gs: Stomatal conductance; Ci: Intercellular CO_2 concentration; A/E: Instant water use efficiency; Ci/Ca: Stomatal limitation ratio. HD: Heading; EF: Early filling; MF: Middle filling.

Supplementary Table 7. Multiple comparisons of yield traits among the clusters of wheat varieties based on CTDs at the three stages for the three growing seasons.

Growing season	Cluster	SN (m^{-2})	TKW (g)	GY (kg/hm^{-2})	BM (kg/hm^{-2})	HI
2016-17	1	681.2±131.2 b	39.8±4.4 a	9121.8±1976.6	21722.9±4195.9	0.42±0.04 a
（Normal）	2	696.8±102.4 b	38.2±5.1 ab	8893±1719.1	21807.8±3482.4	0.4±0.05 a
	3	769.4±120.7 a	35.6±5.2 b	8547.5±2612.4	22880.2±5552	0.37±0.05 b
2017-18	1	376.7±101 a	47.1±4.2	7164.9±1097.5	15827.8±2307.6 a	0.45±0.04
(Freezing)	2	335.7±57.4 b	48.5±4.1	6942±1030.2	15291.5±2149.9 ab	0.46±0.05
	3	346.1±78.9 ab	47.9±5.1	6908.9±929.7	14724.8±2189.6 b	0.47±0.04
2018-19	1	401.4±73	48±4	7379.8±1171.5	14322.2±2298.3	0.51±0.04 a
(Drought)	2	432.4±90.2	47.3±4.8	7143.1±1438.1	14640.1±3079.5	0.48±0.04 b
	3	412.2±102.4	48.7±5.1	7065.4±1720.7	14131.5±3756.6	0.5±0.04 ab

Note: the data was shown as the Mean ± standard deviation. the clustering was conducted with the CTD at the three growth stages for each growing season using the Ward method. Then multiple comparison was conducted among different clusters using the Duncan method, different lowercase letters indicated the differences significant at the $P < 0.05$ level. SN: Spike number per m^2; TKW: 1000-kernel weight; GY: Grain yield; BM: Biomass; HI: Harvest index. HD: Heading; EF: Early filling; MF: Middle filling.

Supplementary Table 8. Clustering based on canopy structural traits for the three growing seasons and multiple comparisons of the traits.

Growing season	Cluster	Numbers	WFL（cm）	LFL （cm）	PH（cm）	PL（cm）	DSL(cm)	SL(cm)	CTD-HD(℃)	CTD-EF(℃)	CTD-MF(℃)
2016-17	1	14	1.7±0.17 b	23±3.6 a	99.5±13.6 a	35.9±1.9 a	16.8±3.2 a	9.5±0.93 ab	-3.8±1.2 b	-2.5±1.2	-4.7±0.69 b
（Normal）	2	94	1.9±0.18 a	20.1±2.2 b	86.1±7 b	28.3±2.3 b	10.9±1.9 b	9.2±0.92 b	-3.0±1.7 ab	-2.2±1.5	-3.6±1.7 a
	3	78	2.0±0.2 a	18.9±2.1 c	82.5±6.6 b	25.3±2.3 c	8.6±1.9 c	9.8±0.73 a	-2.6±1.4 a	-1.7±1.3	-3.0±1.8 a
2017-18	1	7	1.8±0.21 b	20.1±2.6 ab	86.6±7.4 ab	28.2±4.1 a	11.0±3.4 a	9.4±0.92	-4.7±0.82 c	-3.6±1.0 c	-4.9±0.87 b
(Freezing)	2	74	2.0±0.22 a	20.4±2.7 a	87.3±12.4 a	28.1±4.1 a	10.6±3.3 ab	9.5±0.78	-3±0.69 b	-1.7±0.61 b	-4.4±0.86 c
	3	98	2.0±0.16 a	19.3±2.2 b	84±6.6 b	26.8±2.6 b	9.7±2.1 b	9.6±0.9	-1.5±0.64 a	-0.9±0.63 a	-1.7±1.0 a
2018-19	1	9	1.6±0.1 b	18.5±2 a	96.3±12.2 c	34.2±2.4 a	15.5±3.8 a	9±1.1 a	-1.4±0.74	-5.1±1.0 a	-2.9±0.83 b
(Drought)	2	79	1.8±0.18 a	15.5±2 b	77.9±7 b	24.8±2.4 b	8.5±2.1 b	8.4±0.89 b	-1.6±0.93	-5.6±0.64 b	-1.9±1.0 a
	3	98	1.8±0.19 a	17.1±2.6 a	69.1±5.6 a	21±2.5 c	5.4±1.6 c	8.2±0.81 b	-1.4±1.3	-5.8±0.8 b	-1.8±1.1 a

Note: the data was shown as the Mean ± standard deviation. The clustering was conducted with the canopy structural traits for each growing season using the Ward method. The multiple comparison was conducted among different clusters using the Duncan method, different lowercase letters indicated the differences significant at the $P < 0.05$ level. Width of flag leaf; LFL: Length of flag leaf; SL: Spike length; PH: Plant height; PL: Peduncle length; DSL: Distance of spike to leaves; HD: Heading; EF: Early filling; MF: Middle filling; CTD-HD, CTD-EF and CTD-MF: CTD at the heading, early grain filling and middle grain filling stage, respectively.

Supplementary Table 9. Multiple comparisons of photosynthetic traits among the clusters of wheat varieties based on canopy structural traits.

Growing season	Stages	Cluster	A (μmol m^{-2} s^{-1})	Gs (μmol m^{-2} s^{-1})	Ci (μmol mol^{-1})	E (mol m^{-2} s^{-1})	A/E	Ci/Ca
2016-17(Normal)	HD	1	20.4±3	0.3±0.08 b	237.6±32.3	3.9±0.82	5.4±1	0.64±0.08
		2	21.6±2.4	0.34±0.07 ab	247.4±24.1	4±0.64	5.4±0.92	0.66±0.06
		3	21.5±2	0.34±0.07 a	250.3±20.3	4±0.57	5.5±0.75	0.67±0.05
	EF	1	18.9±2.4	0.24±0.06 b	222.5±27.1	4.1±0.7 b	4.8±1.1 a	0.6±0.08
		2	19.6±2.3	0.26±0.06 ab	232.1±23.2	4.7±0.89 a	4.3±0.79 b	0.62±0.06
		3	19.6±2.5	0.27±0.06 a	236.3±23.3	4.9±0.73 a	4.1±0.7 b	0.63±0.06
	MF	1	11.3±4.1	0.28±0.1	306.3±26.7 b	4.1±1.2	4.8±1.1 a	0.79±0.06 b
		2	10.3±4.7	0.3±0.08	319.4±41.2 ab	4.1±0.87	4.3±0.79 b	0.81±0.1 ab
		3	9.8±4	0.33±0.08	329.7±30 a	4.1±0.94	4.1±0.7 b	0.84±0.07 a
2017-18(Freezing)	HD	1	21.7±2.4	0.33±0.07	245.2±18.4 b	4.2±0.59 a	5.2±0.51 b	0.65±0.05 b
		2	21.8±2.3	0.34±0.09	240.4±30.2 b	4.1±0.75 a	5.4±0.88 ab	0.65±0.08 b
		3	21.2±2.2	0.34±0.06	254.1±21.3 a	3.8±0.51 b	5.7±1 a	0.68±0.05 a
	EF	1	18.7±2 b	0.26±0.06	233.5±20.5	4.4±0.76 b	4.3±0.75 a	0.63±0.06
		2	18.9±2.3 b	0.26±0.05	237.1±24.8	4.4±0.75 b	4.4±1.1 a	0.63±0.07
		3	20.6±2.3 a	0.27±0.06	230.6±25.4	5.2±0.72 a	4±0.57 b	0.61±0.07
	MF	1	12.5±5.2 a	0.27±0.07 b	297.1±45.1 c	3.7±1 b	4.3±0.75 a	0.76±0.1 c
		2	10.8±3.4 b	0.34±0.08 a	325.2±23 b	4.2±0.8 a	4.4±1.1 a	0.83±0.05 b
		3	8±2.8 c	0.33±0.08 a	342.1±16.7 a	4.4±0.82 a	4±0.57 b	0.87±0.04 a
2018-19((Drought)	HD	1	26.5±3.7	7.5±1.5 b	0.54±0.1 b	0.28±0.11 b	3.6±0.56	215.1±40.6 b
		2	26.5±4.2	8±1.6 ab	0.63±0.14 a	0.39±0.17 a	3.4±0.42	252±56.2 a
		3	28.4±3.8	8.7±1.5 a	0.67±0.1 a	0.45±0.14 a	3.3±0.41	269.1±40.6 a
	EF	1	22.3±3.8 c	5.3±1.6 b	0.69±0.06	0.34±0.09 b	4.7±2.2 a	274.1±24.1
		2	26.6±4.8 b	7±1.4 a	0.71±0.04	0.45±0.1 a	3.8±0.49 b	285.4±15.7
		3	29.1±3 a	7.9±1.4 a	0.71±0.04	0.49±0.09 a	3.8±0.55 b	284.6±17.2
	MF	1	22.3±2.3	5±1.2	0.69±0.04	0.33±0.07	4.6±1	271±15
		2	22.3±2.2	4.7±0.8	0.69±0.04	0.34±0.06	4.9±0.72	270.1±17.6
		3	22.5±2.2	4.7±1	0.69±0.05	0.34±0.08	4.9±0.9	270.9±21.2

Note: the data was shown as the Mean ± standard deviation. the clustering was conducted with the structural traits the growing season using the Ward method. Then multiple comparison was conducted among different clusters for each stage using the Duncan method, different lowercase letters indicated the differences significant at the $P < 0.05$ level. A: Net photosynthetic rate; E: Transpiration rate; Gs: Stomatal conductance; Ci: Intercellular CO_2 concentration; A/E: Instant water use efficiency; Ci/Ca: Stomatal limitation ratio. HD: Heading; EF: Early filling; MF: Middle filling.

Supplementary Table 10. Multiple comparisons of yield traits among the clusters of wheat varieties based on canopy structural traits for each growing season.

Growing season	Cluster	Variety number	SN (m^{-2})	TKW (g)	GY (kg/hm^{-2})	BM (kg/hm^{-2})	HI
2016-17 （Normal）	1	14	769.4±120.7 a	35.6±5.2 b	8547.5±2612.4	22880.2±5552	0.37±0.05 b
	2	94	696.8±102.4 b	38.2±5.1 ab	8893±1719.1	21807.8±3482.4	0.4±0.05 a
	3	78	681.2±131.2 b	39.8±4.4 a	9121.8±1976.6	21722.9±4195.9	0.42±0.04 a
2017-18 (Freezing)	1	74	656.6±109.3 b	38.6±4.4 ab	8679.4±2012	20578.8±3692.7 b	0.42±0.06 a
	2	7	717.7±108.6 a	37.6±5.4 b	8716.6±1661.5	22191.6±3464.2 a	0.39±0.05 b
	3	98	699.2±126 ab	39.6±4.7 a	9316.9±1987.4	22272.1±4357 a	0.41±0.04 a
2018-19 (Drought)	1	9	493.2±101.3 a	45.1±6.3	7458.1±1870.7	15828±4170.8 a	0.47±0.03 b
	2	79	432.7±74 b	47.7±4.7	7394.5±1252.9	15099.3±2416.7 ab	0.48±0.04 b
	3	98	395.6±87.9 b	48.1±4.2	7079.3±1425.4	13754.1±2963.4 b	0.51±0.04 a

Note: the data was shown as the Mean ± standard deviation. the clustering was conducted with the structural traits for each growing season using the Ward method. The multiple comparison was conducted among different clusters using the Duncan method, different lowercase letters indicated the differences significant at the $P < 0.05$ level. SN: Spike number per m^2; TKW: 1000-kernel weight; GY: Grain yield; BM: Biomass; HI: Harvest index.
