# Supplementary material for: Field Evaluation of Wheat Varieties Using Canopy Temperature Depression in Three Different Climatic Growing Seasons
Source: Plants (Basel). 2022 Dec 12;11(24):3471. doi: 10.3390/plants11243471 (PMC9785455; doi:10.3390/plants11243471)
Supplement: Supplementary file 1 [file plants-11-03471-s001.zip › supplementary/Supplementary Figure-CTD-20221105.pdf]

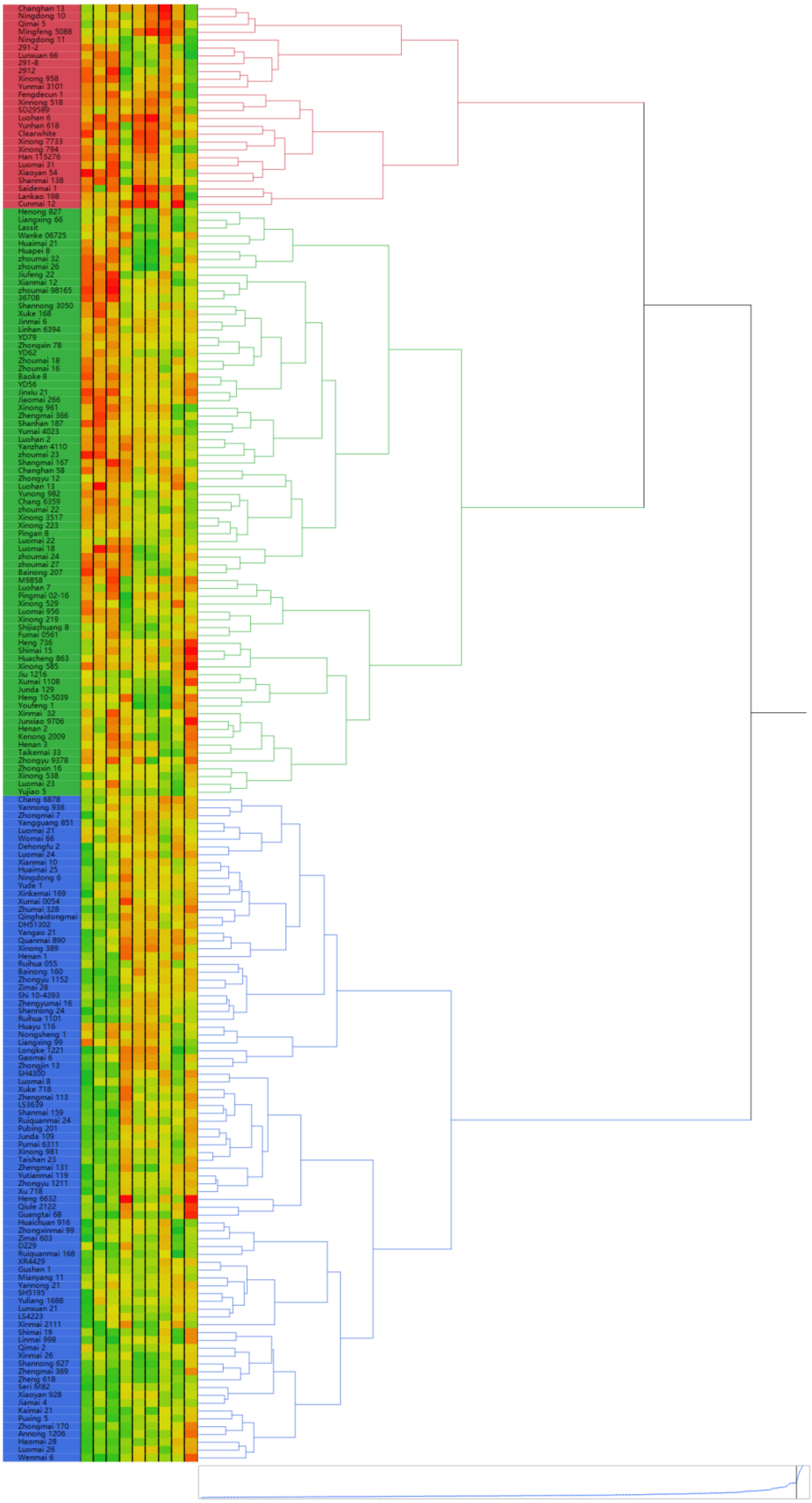

Supplementary Figure 1. The clustering was conducted with the CTD at the three growth stages and structural traits in 2016-17 (normal) growing season using the Ward method.

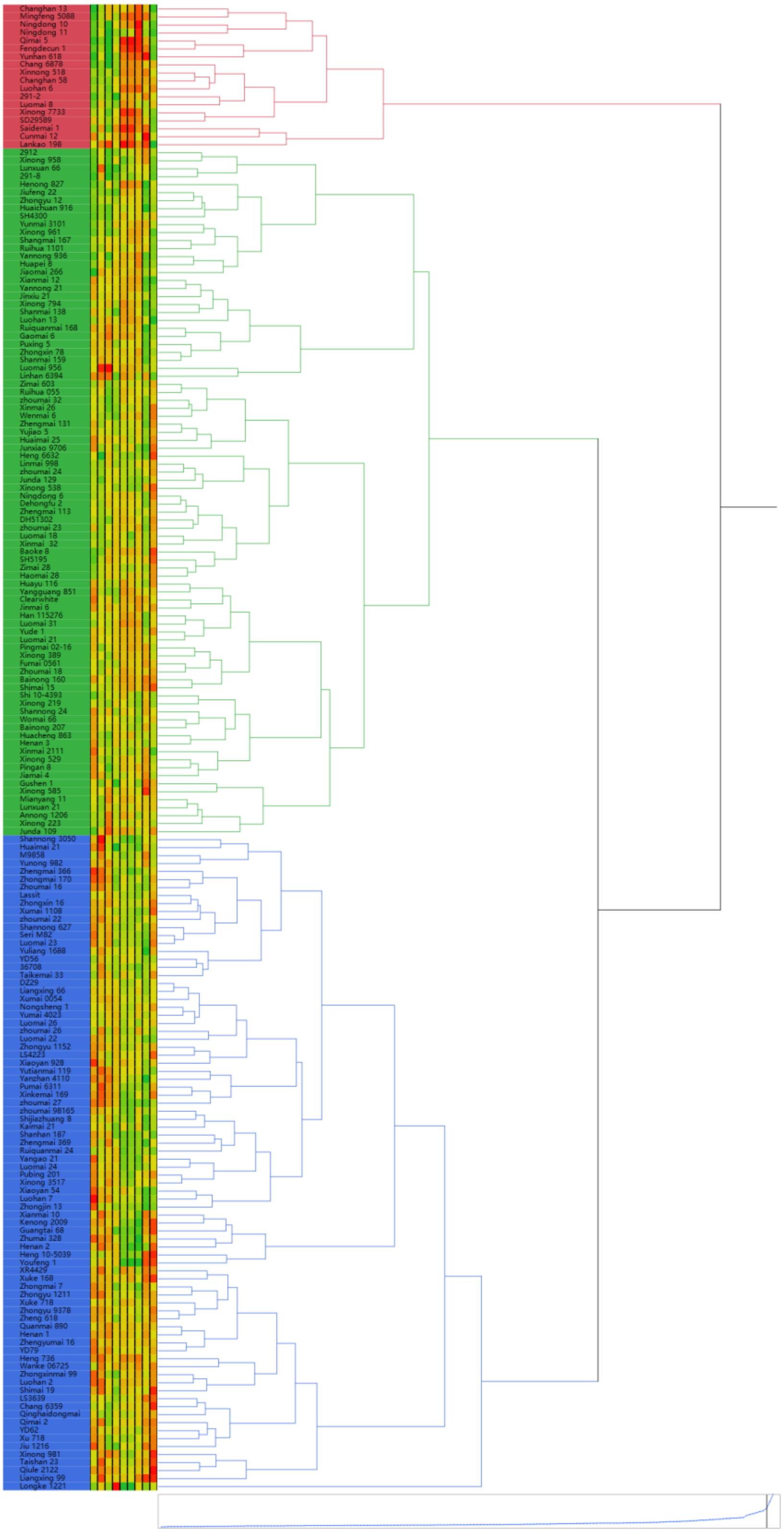

Supplementary Figure 2. The clustering was conducted with the CTD at the three growth stages and structural traits in 2017-18 (freezing) growing season using the Ward method.

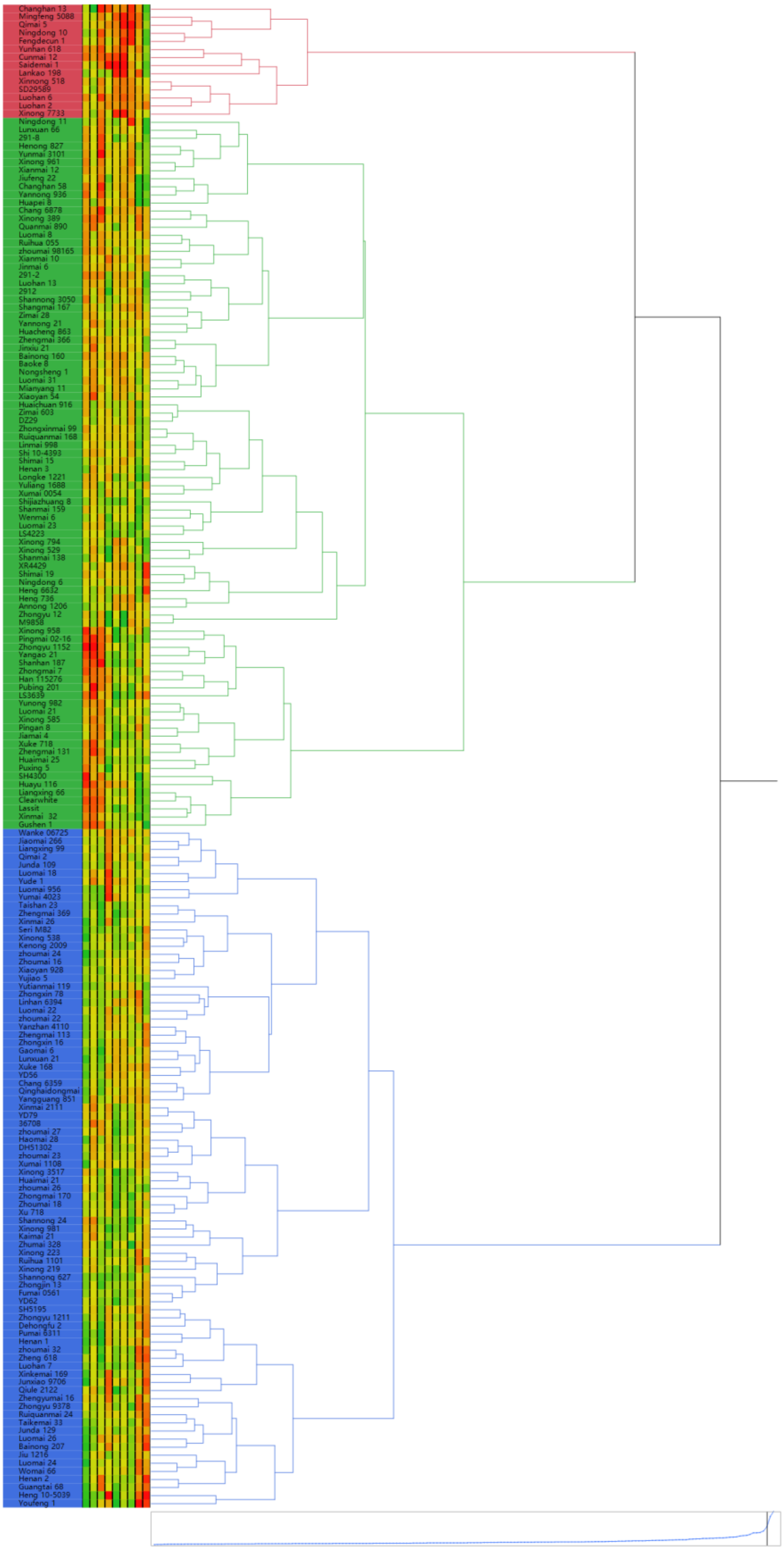

Supplementary Figure 3. The clustering was conducted with the CTD at the three growth stages and structural traits in 2018-19 (drought) growing season using the Ward method.
